# Ten-year trends in epidemiology and outcomes of pediatric kidney replacement therapy in Europe: data from the ESPN/ERA-EDTA Registry

**DOI:** 10.1007/s00467-021-04928-w

**Published:** 2021-01-22

**Authors:** Marjolein Bonthuis, Enrico Vidal, Anna Bjerre, Özlem Aydoğ, Sergey Baiko, Liliana Garneata, Isabella Guzzo, James G. Heaf, Timo Jahnukainen, Marc Lilien, Tamara Mallett, Gabriel Mirescu, Elena A. Mochanova, Eva Nüsken, Katherine Rascher, Dimitar Roussinov, Maria Szczepanska, Michel Tsimaratos, Askiti Varvara, Enrico Verrina, Bojana Veselinović, Kitty J. Jager, Jérôme Harambat

**Affiliations:** 1 ESPN/ERA-EDTA Registry, Amsterdam UMC, University of Amsterdam, Department of Medical Informatics, Amsterdam Public Health Research Institute, Meibergdreef 9, Amsterdam, The Netherlands; 2 Division of Pediatrics, Department of Medicine, University of Udine, Udine, Italy; 3 Division of Pediatric and Adolescent Medicine, Oslo University Hospital, Oslo, Norway; 4 Department of Pediatric Nephrology, 19 Mayis University Medical School, Samsun, Turkey; 5 Department of Pediatrics, Belarusian State Medical University, Minsk, Belarus; 6 Department of Internal Medicine and Nephrology “Dr Carol Davila” Teaching Hospital of Nephrology, “Carol Davila” University of Medicine and Pharmacy, Bucharest, Romania; 7 Nephrology and Dialysis Unit, Pediatric Subspecialties Department, Institute for Scientific Research, Bambino Gesù Children’s Hospital, Rome, Italy; 8 Department of Medicine, Zealand University Hospital, Roskilde, Denmark; 9 Department of Pediatric Nephrology and Transplantation, New Children’s Hospital, University of Helsinki and Helsinki University Hospital, Helsinki, Finland; 10 Wilhelmina Children’s Hospital, University Medical Center, Utrecht, The Netherlands; 11 Department of Paediatric Nephrology, Royal Belfast Hospital for Sick Children, Belfast, UK; 12 Department of Paediatric Nephrology, Royal Bristol Hospital for Children, Bristol, UK; 13 Department of Nephrology and Internal Medicine, “Carol Davila” University of Medicine and Pharmacy, Bucharest, Romania; 14 Department of Nephrology, “Dr. Carol Davila” Teaching Hospital of Nephrology, Bucharest, Romania; 15 Department of Kidney Transplantation, Russian Children’s Federal Clinical Hospital of Pirogov Russian National Research Medical University, Moscow, Russia; 16 Pediatric Nephrology, Department of Pediatrics, Faculty of Medicine and University Hospital Cologne, University of Cologne, Cologne, Germany; 17 QiN-Group, Department of Medicine II, Faculty of Medicine and University Hospital Cologne, University of Cologne, Cologne, Germany; 18 SBAL Pediatric Diseases, Nephrology and Hemodialysis Clinic, Department of Pediatrics, Medical University of Sofia, 1606 Sofia, Bulgaria; 19 Department of Pediatrics, Faculty of Medical Sciences in Zabrze, Medical University of Silesia in Katowice, Katowice, Poland; 20 Department of Multidisciplinary Pediatrics, Pediatric Nephrology Unit, Assistance Publique des Hôpitaux de Marseille, Marseille, France; 21 Nephrology Unit, Kyriakou Children’s Hospital, Athens, Greece; 22 Dialysis Unit, Department of Pediatrics, IRCCS Giannina Gaslini, Genoa, Italy; 23 Nephrology Department, University Children’s Hospital, Belgrade, Serbia; 24 Department of Pediatrics, Bordeaux University Hospital, Bordeaux Population Health Research Center UMR 1219, University of Bordeaux, Bordeaux, France

**Keywords:** Dialysis, Peritoneal dialysis, Hemodialysis, Transplantation, Pediatrics, Epidemiology, ESPN/ERA-EDTA Registry

## Abstract

**Background:**

For 10 consecutive years, the ESPN/ERA-EDTA Registry has included data on children with stage 5 chronic kidney disease (CKD 5) receiving kidney replacement therapy (KRT) in Europe. We examined trends in incidence and prevalence of KRT and patient survival.

**Methods:**

We included all children aged <15 years starting KRT 2007–2016 in 22 European countries participating in the ESPN/ERA-EDTA Registry since 2007. General population statistics were derived from Eurostat. Incidence and prevalence were expressed per million age-related population (pmarp) and time trends studied with JoinPoint regression. We analyzed survival trends using Cox regression.

**Results:**

Incidence of children commencing KRT <15 years remained stable over the study period, varying between 5.5 and 6.6 pmarp. Incidence by treatment modality was unchanged over time: 2.0 for hemodialysis (HD) and peritoneal dialysis (PD) and 1.0 for transplantation. Prevalence increased in all age categories and overall rose 2% annually from 26.4 pmarp in 2007 to 32.1 pmarp in 2016. Kidney transplantation prevalence increased 5.1% annually 2007–2009, followed by 1.5% increase/year until 2016. Prevalence of PD steadily increased 1.4% per year over the entire period, and HD prevalence started increasing 6.1% per year from 2011 onwards. Five-year unadjusted patient survival on KRT was around 94% and similar for those initiating KRT 2007–2009 or 2010–2012 (adjusted HR: 0.98, 95% CI:0.71–1.35).

**Conclusions:**

We found a stable incidence and increasing prevalence of European children on KRT 2007–2016. Five-year patient survival was good and was unchanged over time. These data can inform patients and healthcare providers and aid health policy makers on future resource planning of pediatric KRT in Europe.

**Supplementary Information:**

The online version contains supplementary material available at 10.1007/s00467-021-04928-w.

## Introduction

After decades of continuous growth, the incidence of kidney replacement therapy (KRT) for stage 5 chronic kidney disease (CKD 5) in European adult patients has slightly declined during the last decade, whereas the increase in prevalence has started to slow down and patient survival has improved [1].

CKD 5 etiologies in children are notably different from those in adults, as kidney failure at pediatric age is mostly caused by congenital or hereditary disorders rather than by diabetes or hypertension which are the most common causes in adults [2, 3]. Despite major improvements in pediatric nephrology care over the past 30–40 years, mortality on KRT remains 30 times higher compared to healthy peers [4, 5]. Moreover, improved patient care has resulted in the acceptance of more challenging patients into pediatric KRT programs, including very young patients and patients with more comorbidities [4].

For pediatric patients from the USA, the incidence of KRT decreased by 21% since 2004 after a long period of increasing incidence rates, whereas the prevalence was stable over the last decade [6]. Furthermore, in Australia and New Zealand, the incidence rate varied widely from year to year, but remained overall stable over the past 20 years. On the other hand, the prevalence of pediatric KRT in Australia increased, whereas there was no trend in New Zealand [7]. Data on trends in pediatric KRT are important to inform patients and healthcare providers and to aid healthy policy makers on future resource planning of pediatric KRT in Europe. Earlier European data showed a rise in KRT incidence in the 1980s which remained stable thereafter, while the prevalence of KRT among patients aged less than 20 years tripled from 1980 to 2000 [8]. However, more recent data on the incidence and prevalence of pediatric KRT in Europe have only been reported over a short time frame [4], and trends were not studied.

With 10 consecutive years of data collection on European pediatric KRT patients within the ESPN/ERA-EDTA Registry, we are now able to report on trends in the incidence, prevalence, treatment modality, and patient survival from 2007 to 2016.

## Methods

**Fig. 1:**
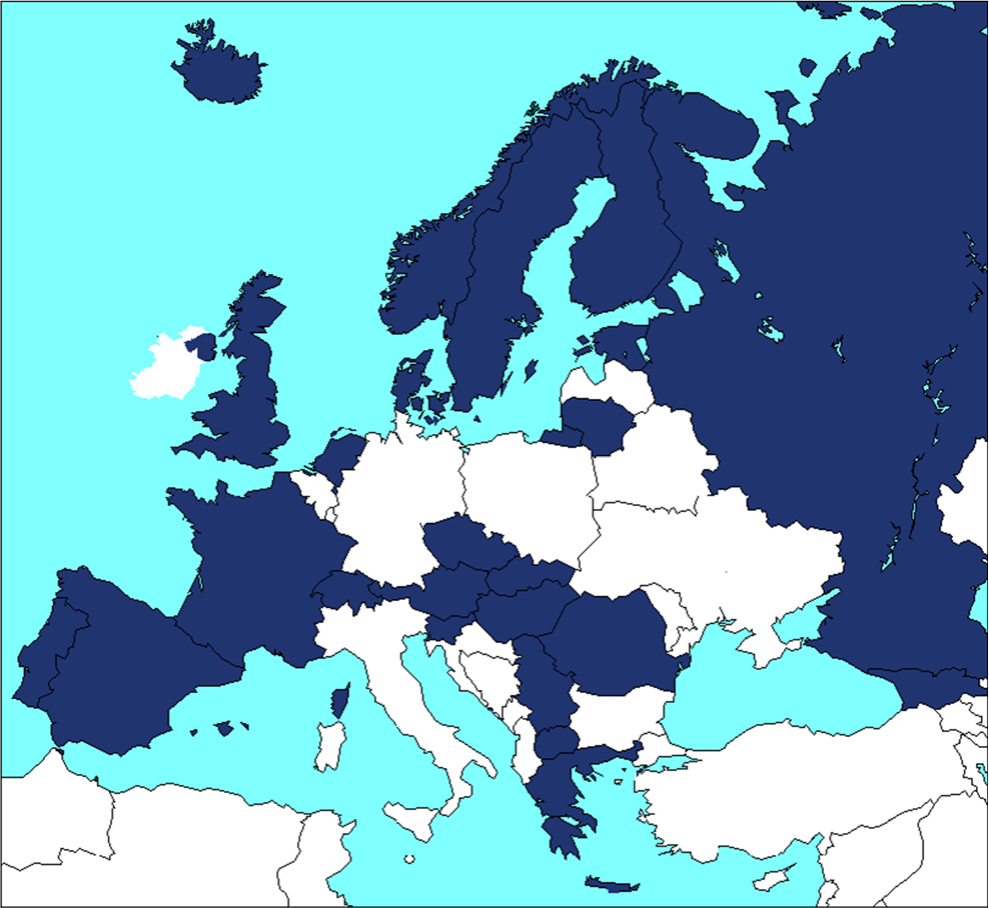
European countries included in the study

Patient data were extracted from the ESPN/ERA-EDTA Registry, a population-based registry that collects data on individual pediatric patients on KRT in Europe on an annual basis [9, 10]. Currently, 36 countries participate in the Registry. Most countries report information from pediatric centers only. As older children (15–19 years of age) may be treated in adult centers, they might not be included in the ESPN/ERA-EDTA Registry, possibly leading to an underestimation of incidence and prevalence figures. Therefore, for this study, we included data of patients initiating KRT < 15 years of age from the 22 countries contributing with data on all treatment modalities for the entire period from January 1, 2007, to December 31, 2016 (Fig. 1). For comparison reasons, we also report incidence and prevalence rates for the other countries, but these numbers are not included in the trend analyses.

Data were categorized as follows: age at initiation of KRT (for incidence) or at the 31st of December of each year (for prevalence) (0 < 5 years, 5 < 10 years, 10 < 15 years), sex (male or female), treatment modality (hemodialysis (HD), peritoneal dialysis (PD), or kidney transplantation), cause of CKD 5 (categorized according to the ERA-EDTA coding system adapted for children) [3], calendar year (2007 to 2016).

Incidence rates per million age-related population (pmarp) were calculated as the number of new cases on KRT in each calendar year divided by the mid-year age-related general population. General population characteristics were extracted from the Eurostat database [11].

Prevalence was calculated as the number of cases being alive and treated with KRT on the 31st of December of each calendar year and expressed as pmarp using the mid-year general population.

To evaluate time trends in the observed incidence and prevalence, the JoinPoint regression program provided by the Surveillance Research Program of the US National Cancer Institute was used. The annual percentage change (APC) was computed using Poisson regression provided by the JoinPoint regression program, as described previously by Kramer et al. [12]. In short, the program fits a series of joint straight lines on a log scale to the trends in observed rates. Line segments are joined at points called joinpoints, and each joinpoint denotes a statistically significant change in trend. The APC (i.e., the slope of the line segment) was calculated with the observed rate as outcome variable and year as explanatory variable, in order to describe and test the significance of the trends.

Patient survival probabilities were defined as the probability that a person has survived up to a specific time point, e.g., 1, 2, or 5 years after commencing KRT or after receiving a first kidney transplant, and causes of death were classified according to the ERA-EDTA Registry coding system, whereas cardiac failure, cardiac arrest/sudden death, myocardial ischemia and infarction, and cerebro-vascular accidents were combined as cardiovascular mortality [3]. Patients were followed from the first day of KRT (patient survival on KRT) or from the day of first kidney transplant (patient survival after transplantation) until death, lost to follow-up or end of study, whichever occurred first.

The annual number of kidney transplants performed (pmarp) between 2007 and 2016 was calculated, as well as the 1-, 2 -, and 5-year graft survival, defined as being alive with a functioning kidney transplant at 1, 2, or 5 years after transplantation, respectively.

The Cox proportional hazard regression model was used to calculate patient and graft survival probabilities, while accounting for confounders. To compare adjusted 5-year survival between two periods of initiating KRT (2007–2009 and 2010–2012), adjusted hazard ratios were calculated using Cox proportional hazard regression models. Adjustments were made for the confounding effects of country, sex, cause of CKD 5, and age and treatment modality at start of KRT.

Analyses were performed using SAS 9.4 statistical software package (SAS Institute, Cary, NC, USA) and JoinPoint version 4.7.0.0.

Two-sided *P* values of 0.05 were considered statistically significant.

## Results

### Trends in incidence

A total of 4459 patients aged 0–14 years from 22 European countries initiated KRT between 2007 and 2016. The yearly incidence of patients under 15 years commencing KRT by country fluctuated considerably from year to year (Table 1). The overall KRT incidence in Europe ranged between 5.5 pmarp and 6.6 pmarp, corresponding to 401 and 482 patients initiating KRT annually. There was no trend in the overall incidence in Europe from 2007 to 2016 (APC: − 0.8, 95% CI: − 2.5 to 1.0).

**Table 1:** Incidence of KRT (pmarp) among 0–14-year-old children by country and year

Country	Year										
	2007	2008	2009	2010	2011	2012	2013	2014	2015	2016	Average
Albania*				3.1	1.6	3.4	1.7	0.0	9.3	9.5	4.1
Austria	7.0	5.5	10.4	4.0	7.3	4.1	1.6	6.5	4.0	6.4	5.7
Belarus*		5.6	4.2	5.0	4.9	4.2	5.4	5.3	3.9	3.8	4.7
Belgium*	7.8	2.8	3.3	6.5	3.7			2.6			4.4
Bosnia and Herzegovina*					8.2	3.3	8.2	5.2	3.7	1.8	5.1
Bulgaria*			4.1	4.1	3.1	5.1	2.0	3.0	9.0	2.0	4.2
Croatia*		8.8	8.9	4.5	6.1	7.8	6.3	6.4	4.9	11.5	7.2
Cyprus*							0.0	7.2	21.5	14.3	10.8
Czech Republic	6.1	2.7	4.0	4.6	6.5	2.6	5.1	5.7	5.0	4.3	4.7
Denmark	9.9	6.9	16.9	7.0	5.0	7.1	8.2	7.2	5.2	5.2	7.9
Estonia	0.0	5.0	0.0	0.0	4.9	0.0	0.0	0.0	9.5	0.0	1.9
Finland	12.3	4.5	6.7	9.0	6.8	9.0	9.0	12.3	14.5	3.4	8.7
France	6.6	6.4	6.3	6.3	4.9	5.1	6.5	5.4	7.4	6.7	6.2
Georgia*							2.6	12.7	7.4	5.6	7.1
Germany CERTAIN*^1^				3.6	2.0	1.2	0.6	0.6	0.2	0.9	1.3
Germany KfH*^2^								2.5	2.8	3.9	3.1
Greece	8.0	6.8	8.0	4.3	3.1	6.8	1.9	5.7	5.7	2.6	5.3
Hungary	3.3	4.0	9.4	4.8	5.5	7.7	7.0	6.3	14.7	6.3	6.9
Iceland	15.2	0.0	0.0	15.0	15.1	0.0	15.0	0.0	0.0	0.0	6.0
Ireland*										11.9	11.9
Italy*^3^	4.5	4.5	4.7	3.2	3.6	4.7	3.9	4.3	3.5	3.0	4.0
Latvia*								3.4	3.3		3.4
Lithuania	3.9	2.0	8.4	0.0	2.2	6.8	6.9	0.0	9.4	2.4	4.2
Malta*				0.0	0.0	0.0	0.0	0.0	0.0	15.5	2.2
Moldova*					1.7	1.7					1.7
Montenegro*	0.0	24.5	0.0								8.2
North Macedonia	7.9	0.0	2.7	2.8	2.8	0.0	5.7	0.0	2.9	0.0	2.5
the Netherlands	7.5	7.9	8.2	6.9	4.8	6.2	5.9	6.3	7.1	6.4	6.7
Norway	5.5	7.7	10.9	4.3	10.8	5.4	5.4	9.7	11.8	5.3	7.7
Poland*	6.9	6.6	6.5	6.9	6.0	4.3	4.9	7.0	4.0		5.9
Portugal	9.1	7.3	10.5	5.0	9.5	12.2	4.6	8.6	6.8	4.8	7.9
Romania	2.3	5.2	5.0	4.7	6.3	4.7	4.5	3.2	3.3	3.9	4.3
Russia	4.4	3.7	3.4	3.9	3.5	4.8	3.6	4.9	3.5	3.3	3.9
Serbia	4.4	11.5	3.6	2.8	4.8	2.9	5.8	5.9	4.9	4.9	5.2
Slovakia	4.6	3.5	6.0	3.6	4.8	2.4	6.0	7.2	3.6	2.4	4.4
Slovenia	7.1	14.2	3.5	10.4	3.4	3.4	6.7	19.8	3.3	9.8	8.2
Spain	6.7	7.4	8.3	5.4	7.1	5.8	6.5	6.2	6.0	7.1	6.6
Sweden	7.1	7.1	9.7	12.2	8.3	13.1	8.6	9.6	5.9	8.6	9.0
Switzerland*^4^		5.1	4.3	3.4	8.4	11.7	3.3	4.9	9.7	7.2	6.4
Turkey*^5^				3.0	3.1	3.1	3.2	4.1	3.2	3.3	3.3
Ukraine*				0.9	3.1	4.9	3.6	3.9	2.9	4.0	3.3
UK	11.0	11.0	9.6	8.2	7.8	9.6	9.5	10.6	10.7	8.9	9.7
Total	6.5	6.3	6.6	5.6	5.5	6.1	5.8	6.4	6.3	5.5	6.1

*KRT*, kidney replacement therapy; *pmarp*, per million age-related population

^*^Not included in the total incidence

^1^Data are based on transplantation patients only; therefore, the numbers are an underestimation of the true incidence in Germany. The coverage ranged from 12 out of 20 centers in 2010 to 18 out of 20 centers in 2016. Each year around 120 patients under the age of 21 years are transplanted, of which 16% pre-emptively

^2^Dialysis patients only. In 2015, data from one dialysis center was not included, and the incidence of dialysis patients is an underestimation of the true incidence

^3^(Pre-emptive) transplantation patients are not included;

^4^In 2013, not all patients provided informed consent resulting in an underestimation of the true incidence;

^5^For 2010, data are based on patients aged 0–15 years. The incidence is an underestimation of the true incidence

**Fig. 2:**
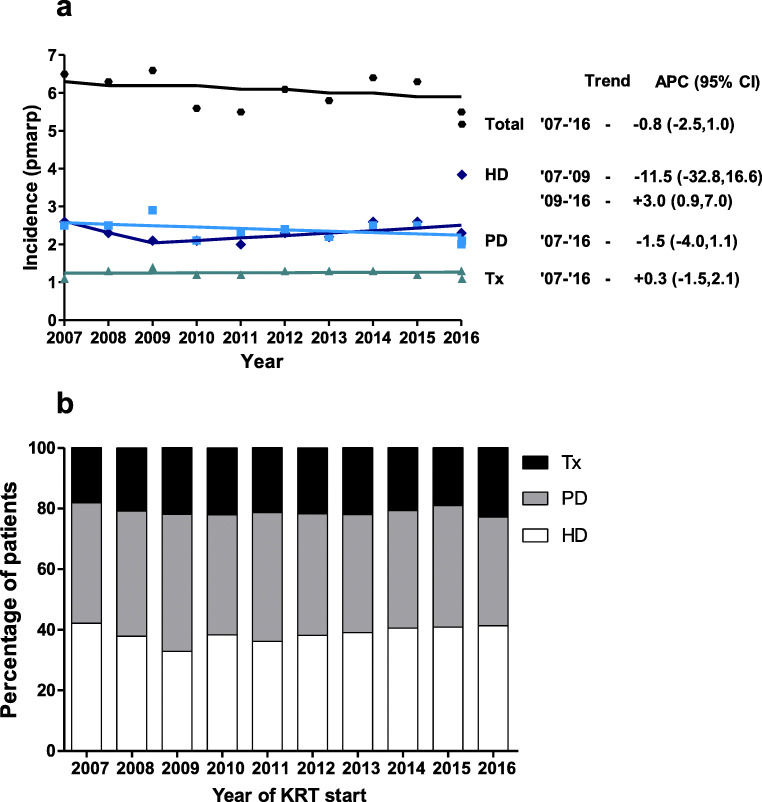
Incidence of KRT by treatment modality and year. **a** Trends in the incidence rate of pediatric KRT by treatment modality (pmarp). Trends were calculated by the APC and its 95% confidence interval*.* **b** Distribution of first KRT modality by year. Abbreviations: KRT, kidney replacement therapy; pmarp, per million age-related population; APC, annual percentage change; HD, hemodialysis; PD, peritoneal dialysis; Tx, pre-emptive kidney transplantation

The incidence of HD tended to decrease from 2.6 pmarp in 2007 to 2.0 pmarp in 2011 and to gradually increase back to 2.6 pmarp in 2014 and 2015, but these changes were not statistically significant (Fig. 2a). The incidence of PD fluctuated between 2.0 pmarp and 3.0 pmarp, but there was no significant trend between 2007 and 2016 (APC: − 1.5, 95% CI: − 4.0 to 1.1). The pre-emptive transplantation rate showed a more stable pattern and was around 1.0 pmarp over the entire period (Fig. 2a). The distribution of first treatment modality was stable, with 80% of patients commencing KRT on dialysis (HD and PD both 40%), while 20% received a pre-emptive kidney transplant (Fig. 2b).

**Fig. 3:**
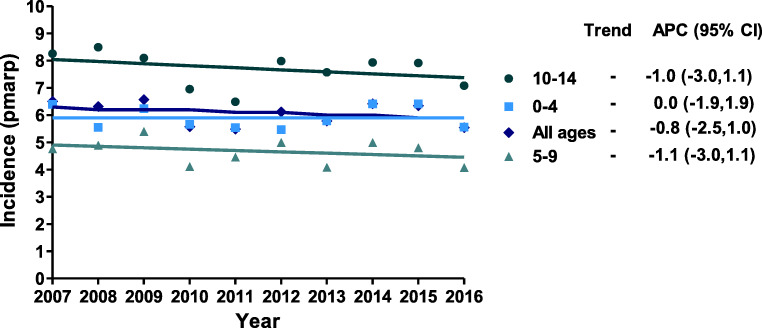
Incidence of KRT (pmarp) by age group and year. Trends were calculated by the APC and its 95% confidence interval. Abbreviations: KRT, kidney replacement therapy; pmarp, per million age-related population; APC, annual percentage change

Figure 3 shows the incidence rate by age group for all countries combined. The incidence rate was the highest among 10–14-year-old patients (ranging from 7.1 to 8.5 pmarp) and the lowest among patients aged 5–9 years (ranging from 4.1 to 5.4 pmarp), while it ranged from 5.5 to 6.4 pmarp in the youngest patients (< 5 years). Although the incidence rate by age group fluctuated, there were no clear trends in incidence rate according to age group.

A statistically significant decrease in incidence rate was observed for patients with congenital anomalies of the kidney and urinary tract (CAKUT) (APC: − 3.3, 95% CI: − 5.5 to − 1.0) and metabolic disorders (APC: − 8.1, 95% CI: − 14.3 to − 1.5) leading to CKD 5, whereas the incidence rate of unknown or missing causes of CKD 5 increased significantly by 5.2% annually (95% CI: 1.1 to 9.4) between 2007 and 2016. Although not statistically significant, we observed a tendency towards decreasing numbers of patients commencing KRT due to vasculitis and hemolytic uremic syndrome (HUS). The incidence of KRT due to other causes remained stable over the study period (Table 2).

**Table 2:** Incidence (pmarp) by cause of stage 5 CKD and year

Cause of CKD 5	Year										APC
	2007	2008	2009	2010	2011	2012	2013	2014	2015	2016	
Glomerulonephritis	1.0	1.0	1.0	0.8	1.0	0.8	0.8	0.8	0.9	0.9	−1.0 (−4.0;+2.0)
CAKUT	2.7	2.4	2.7	2.4	2.2	2.6	2.2	2.2	2.3	1.7	−3.3 (−5.5; −1.0)*
Cystic kidneys	0.7	0.8	0.5	0.5	0.8	0.7	0.8	0.9	0.9	0.6	+2.0 (−2.8;+7.1)
Hereditary nephropathy	0.4	0.4	0.5	0.3	0.4	0.4	0.6	0.5	0.4	0.5	+1.8(−2.7;+6.5)
Ischemic kidney failure	0.2	0.2	0.1	0.1	0.1	0.1	0.1	0.1	0.1	0.1	−5.0 (−11.7;+2.1)
HUS	0.3	0.4	0.3	0.3	0.2	0.2	0.2	0.3	0.2	0.2	−4.9 (−9.7;+0.1)
Metabolic disorder	0.2	0.3	0.2	0.2	0.1	0.2	0.2	0.2	0.1	0.1	−8.1 (−14.3;-1.5)*
Vasculitis	0.1	0.2	0.1	0.1	0.0	0.1	0.1	0.1	0.1	0.1	−9.0 (−18.0;+0.8)
Miscellaneous	0.6	0.4	0.6	0.5	0.3	0.5	0.4	0.7	0.8	0.9	+6.1 (−0.8;+13.6)
Missing/unknown	0.4	0.3	0.4	0.4	0.4	0.6	0.6	0.6	0.5	0.5	+5.2 (+1.1;+9.4)*

*pmarp*, per million age-related population; *CKD 5*, stage 5 chronic kidney disease; *CAKUT*, congenital anomalies of the kidney and the urinary tract; *HUS*, hemolytic uremic syndrome

^*^Statistically significant trend

### Trends in prevalence

The prevalence by country and year is shown in Table 3. There were large country differences, but the overall prevalence increased by 1.9% annually (95% CI: 1.5–2.3) from 2120 children (29.5 pmarp) on KRT on December 31st, 2007, to 2675 children on KRT (35.6 pmarp) on December 31st, 2016.

**Table 3:** Prevalence of KRT (pmarp) among 0–14-year-old children by country and year

Country	Year										
	2007	2008	2009	2010	2011	2012	2013	2014	2015	2016	Average
Albania^*^				4.7	4.9	8.5	7.0	7.2	11.1	9.5	7.5
Austria	40.4	41.8	46.3	40.3	43.1	40.1	36.9	38.4	40.4	41.4	40.9
Belarus^*^		19.0	20.5	21.2	22.5	20.8	21.7	23.3	24.8	24.3	22.0
Belgium^*^		57.2	53.7	49.3	46.1			36.6			48.6
Bosnia and Herzegovina^*^					14.8	14.8	18.1	20.9	20.2	20.2	18.2
Bulgaria^*^		17.3	10.7	11.7	10.2	12.5	13.1	8.0	18.0	10.0	12.4
Croatia^*^		29.4	32.5	29.9	30.3	40.6	42.7	36.8	40.6	51.0	37.1
Cyprus^*^							35.5	50.2	64.4	71.7	55.4
Czech Republic	23.8	21.7	22.2	22.5	26.8	22.6	22.9	26.4	28.5	27.5	24.6
Denmark	42.5	40.6	50.8	51.1	49.5	54.0	49.3	43.5	38.4	40.5	46.1
Estonia	10.0	15.1	10.0	4.9	9.8	9.7	9.6	9.6	19.0	18.8	11.7
Finland	92.4	80.6	80.9	82.2	84.4	85.4	85.1	89.3	97.0	80.4	85.8
France	30.1	32.9	35.6	36.0	35.8	35.3	36.9	37.8	39.0	39.0	35.9
Georgia^*^							7.8	18.3	19.1	20.9	16.5
Germany CERTAIN^*1^				20.5	17.6	21.6	16.8	19.2	20.2	22.7	19.8
Germany KfH^*2^								10.8	11.9	14.2	12.3
Greece	29.0	30.2	33.8	35.0	32.0	37.0	32.3	34.0	36.4	34.0	33.4
Hungary	32.9	28.7	33.0	29.3	30.4	33.4	37.8	35.8	38.6	40.7	34.0
Iceland	30.4	30.2	30.1	45.1	60.2	60.3	60.0	59.8	59.9	45.0	48.1
Ireland^*^										75.5	75.5
Italy^*3^	29.7	31.6	31.2	30.3	30.4	31.4	32.0	32.7	31.2	30.7	31.1
Latvia^*^								16.9	16.7		16.8
Lithuania	33.4	34.7	42.0	28.1	24.5	22.7	25.4	18.7	18.8	21.3	27.3
Malta^*^				63.7	64.6	65.0	64.9	64.2	31.6	31.0	55.0
Moldova^*^					6.9	1.7					4.3
Montenegro^*^	8.1	32.7	32.8								24.5
North Macedonia	18.3	16.0	19.1	19.4	22.4	14.2	17.2	11.5	14.4	14.5	16.8
the Netherlands	44.1	43.7	45.9	44.3	43.8	44.0	42.9	42.6	44.1	45.5	44.1
Norway	47.4	44.0	47.0	41.3	46.6	47.5	45.2	49.4	57.9	56.6	48.3
Poland^*^	34.4	34.8	37.7	40.6	40.6	40.8	41.0	40.1	38.7		38.7
Portugal	38.2	40.9	44.9	45.4	50.5	59.6	54.7	57.1	56.3	50.3	49.6
Romania	6.1	9.7	12.1	11.9	13.5	15.2	15.1	13.9	14.0	16.0	12.6
Russia	9.4	11.0	12.0	13.0	14.3	16.3	16.3	18.0	18.5	18.8	14.9
Serbia	24.5	35.6	35.9	31.5	29.7	30.0	29.2	28.3	30.3	29.5	30.4
Slovakia	27.8	26.0	27.5	26.4	25.2	21.6	26.5	21.7	20.5	19.1	24.2
Slovenia	17.8	31.9	31.5	38.1	34.2	37.1	30.0	36.3	22.9	32.5	31.3
Spain	39.7	40.4	42.2	39.2	41.4	39.9	40.3	41.0	41.0	43.3	40.6
Sweden	49.8	46.7	49.8	55.2	54.0	59.4	57.7	63.7	57.1	58.1	55.3
Switzerland^*4^		44.1	42.4	42.2	42.7	48.3	42.1	42.4	42.0	56.6	44.7
Turkey^*^				32.1	16.0	17.4	17.7	13.3	14.3	15.7	18.1
Ukraine^*^				8.3	7.8	10.0	11.6	13.2	12.5	13.4	11.0
UK	53.1	53.7	53.8	52.7	50.6	50.9	50.8	53.5	55.8	55.9	53.1
Total	29.4	30.6	32.4	32.0	32.5	33.4	33.3	34.4	35.3	35.6	32.9

*KRT*, kidney replacement therapy; *pmarp*, per million age-related population

^*^Not included in the total prevalence

^1^Data are based on transplantation patients only; therefore, the numbers are an underestimation of the true prevalence in Germany. The coverage ranged from 12 out of 20 centers in 2010 to 18 out of 20 centers in 2016. Each year around 120 patients under the age of 21 years are transplanted, of which 16% pre-emptively

^2^Dialysis patients only. In 2015, data from one dialysis center was not included, and the prevalence of dialysis patients is an underestimation of the true incidence

^3^(Pre-emptive) transplantation patients are not included; the prevalence is an underestimation of the true prevalence

^4^In 2013 and 2014, not all patients provided informed consent resulting in an underestimation of the true prevalence

^5^For 2010, data are based on patients aged 0–15 years. The prevalence is an underestimation of the true prevalence

**Fig. 4:**
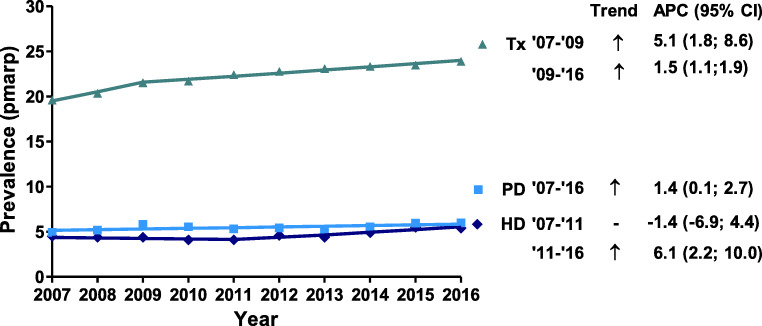
Prevalence of KRT (pmarp) by treatment modality and year. Trends were calculated by the APC and its 95% confidence interval. Abbreviations: KRT, kidney replacement therapy; pmarp, per million age-related population; APC, annual percentage change, HD, hemodialysis; PD, peritoneal dialysis; Tx, kidney transplantation

The prevalence of patients on all three treatment modalities increased during the study period (Fig. 4). For transplantation, the increase was the strongest in the first 3 years, with an annual change of 5.1% (95% CI: 1.8–8.6), whereas the annual increase was 1.5% (95% CI: 1.1–1.9) for the following years. The HD prevalence started to increase from 2011 onwards with 6.1% annually (95% CI: 2.2–10.0), while PD prevalence steadily increased by 1.4% annually (95% CI: 0.1–2.7) between 2007 and 2016.

**Fig. 5:**
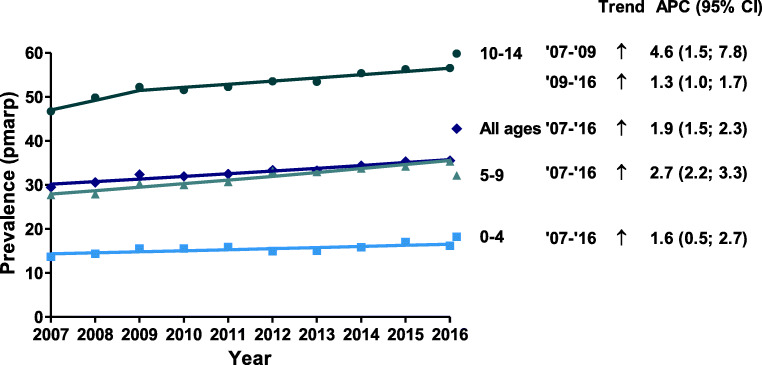
Prevalence of KRT (pmarp) by age group and year. Trends were calculated by the APC and its 95% confidence interval. Abbreviations: KRT, kidney replacement therapy; pmarp, per million age-related population; APC, annual percentage change

For all age groups, the prevalence increased (Fig. 5). The smallest increase was observed for the youngest patients (APC: 1.6%, 95% CI: 0.5–2.7), while the strongest increase was observed for patients aged 10–14 years between the years 2007 and 2009, with an annual increase of 4.6% (95% CI: 1.5–7.8%). In the period thereafter, the prevalence for 10–14-year-old patients increased by 1.3% annually.

### Patient survival on KRT and causes of death

During follow-up, 225 patients died after a median follow-up of 4.1 years (IQR: 1.9–6.4). Overall 1-, 2-, and 5-year patient survival were 97.6% (95% CI: 97.1–98.1), 96.4% (95% CI: 95.8–96.9), and 94.4% (95% CI: 93.6–95.2), respectively. Similar adjusted survival probabilities were found (Table 4). Most patients died from cardiovascular disease (28.9%), followed by infections (22.7%), and for 23.1% of patients, the cause of death was missing or unknown.

**Table 4:** Unadjusted and adjusted KRT patient survival probabilities

Period	Unadjusted patient survival probability (95% CI)			Adjusted patient survival probability (95% CI)		
	1 year	2 years	5 years	1 year	2 years	5 years
2007	97.2 (95.7–98.7)	95.7 (93.9–97.6)	94.1 (92.0–96.3)	98.1 (96.9–99.3)	96.7 (95.4–98.6)	95.8 (93.9–97.9)
2008	96.7 (95.0–98.3)	95.8 (93.9–97.6)	93.4 (91.1–95.7)	97.5 (96.1–98.9)	96.8 (95.2–98.4)	94.9 (92.8–97.1)
2009	97.0 (95.5–98.6)	95.5 (93.6–97.4)	94.2 (92.1–96.3)	97.5 (96.1–98.9)	96.5 (94.8–98.2)	95.4 (93.4–97.5)
2010	96.3 (94.4–98.1)	95.3 (93.2–97.4)	93.7 (91.4–96.1)	98.6 (97.4–99.7)	98.2 (96.8–99.6)	97.5 (95.8–99.3)
2011	98.2 (96.9–99.5)	96.1 (94.2–98.1)	93.4 (91.5–96.4)	98.4 (97.2–99.6)	96.5 (94.7–98.5)	94.6 (92.2–97.1)
2012	96.6 (94.4–98.0)	95.0 (93.0–97.1)		97.1 (95.5–98.7)	96.2 (94.4–98.1)	
2013	97.7 (96.2–99.1)	96.7 (95.3–98.6)		99.4 (98.5–100)	99.2 (98.3–100)	
2014	98.5 (97.4–99.6)	97.7 (96.3–99.0)		99.9 (88.2–100)	99.9 (82.7–100)	
2015	99.6 (99.0–100)			99.8 (84.9–100)		
2007–2016	97.6 (97.1–98.1)	96.4 (95.8–96.9)	94.4 (93.6–95.2)	98.2 (97.8–98.6)	97.3 (96.8–97.8)	95.8 (95.0–96.5)

*Adjusted for fixed values for age at KRT (8.5 years), male sex (59%), glomerulonephritis (15%), CAKUT (38%), hereditary nephropathy (12%), and cystic kidney disease (7%)

For patients commencing KRT in the period 2007–2009, the 5-year unadjusted survival probability was 93.9% (95% CI: 92.6–95.2), whereas it was 93.3% (95% CI: 91.8–94.7) for patients commencing KRT between 2010 and 2012. After adjustment for age, sex, cause of CKD 5, and treatment modality at start, the patient survival at 1, 2 (data not shown), and 5 years did not differ between the periods (adjusted HR 2010–2012 vs. 2007–2009: 0.98, 95% CI: 0.71–1.35). The causes of death differed by period (Table 5). Patients who commenced KRT between 2007 and 2009 died more often from infections (31.3%), whereas cardiovascular disease was more often the cause of death (41.3%) for patients who commenced KRT between 2010 and 2012 (*P* < 0.001).

**Table 5:** Causes of death by period of initiating KRT

Cause of death	Period of initiating KRT N (%)	
	2007–2009	2010–2012
Cardiovascular disease^1^	18 (21.7)	33 (41.3)
Infections^2^	26 (31.3)	10 (12.5)
Malignancies^3^	7 (8.4)	1 (1.3)
Other identified causes^4^	17 (20.5)	11 (13.8)
Unknown/missing	15 (18.1)	25 (31.3)

*KRT*, kidney replacement therapy; *CKD 5*, stage 5 chronic kidney disease

^1^Cardiac arrest/sudden death, fluid overload/pulmonary edema, cerebro-vascular accident, other cause of cardiac failure; ^2^Pulmonary infection (bacterial, viral, parasitic), infections elsewhere, septicemia, generalized viral infection, (bacterial) peritonitis; ^3^Solid tumors, malignancies possibly induced by immunosuppressive therapy; ^4^Hyperkalemia, pulmonary embolus, other hemorrhage, uremia caused by graft failure, chronic obstructive airway disease, accident unrelated to CKD 5 treatment, patient refused further treatment of CKD 5, CKD 5 treatment withdrawn for medical reasons, malignant disease possibly induced by immunosuppressive therapy, malignant disease: solid tumors, other identified cause of death

### Kidney transplantation and graft survival

The overall number of kidney transplants (pre-emptive and non-pre-emptive, deceased and living donor) performed annually was stable and varied between from 3.1 to 3.9 pmarp (APC: − 1.4%, 95% CI: − 2.8–0.1).

Overall unadjusted graft survival was 94.4% (95% CI: 93.5–95.3) at 1 year and 89.1% (95% CI: 87.8–90.5) at 5 years post-transplantation. Graft survival varied by year between 90.8% (95% CI: 87.3–94.4) and 97.0% (95% CI: 94.8–99.2) at 1 year and between 87.1% (95% CI: 83.2–91.2) and 94.1% (95% CI: 91.2–97.0) at 5 years after transplantation, but did not change over time (Table 6).

**Table 6:** Unadjusted and adjusted 1-, 2-, and 5-year graft survival probabilities

Period	Unadjusted graft survival probability (95% CI)			Adjusted graft survival probability (95% CI)*		
	1 year	2 years	5 years	1 year	2 years	5 years
2007	90.8 (87.3–94.4)	89.6 (86.0–93.4)	87.2 (83.2–91.4)	91.2 (87.7–94.9)	90.1 (86.3–94.0)	87.7 (83.5–92.1)
2008	96.5 (94.3–98.8)	96.5 (94.3–98.8)	94.1 (91.2–97.0)	96.8 (94.7–99.0)	96.8 (94.7–99.0)	94.5 (91.6–97.5)
2009	93.3 (90.3–96.4)	90.9 (87.5–94.5)	86.8 (82.7–91.1)	94.6 (91.8–97.5)	92.6 (89.3–96.1)	89.2 (85.0–93.7)
2010	93.2 (90.3–96.2)	91.4 (88.1–94.8)	87.1 (83.2–91.2)	93.2 (90.3–96.3)	91.4 (88.0–94.9)	87.2 (83.1–91.5)
2011	94.2 (91.5–97.1)	92.3 (89.1–95.6)	86.3 (82.2–90.6)	94.6 (91.9–97.4)	92.7 (89.6–96.1)	87.1 (82.9–91.6)
2012	96.3 (94.0–98.7)	95.1 (92.4–97.8)		96.3 (94.0–98.8)	95.1 (92.4–98.0)	
2013	95.3 (92.8–97.8)	94.1 (91.3–97.0)		96.3 (94.0–98.7)	95.4 (92.7–98.1)	
2014	97.0 (94.8–99.2)	95.3 (92.6–98.0)		97.3 (95.2–99.5)	95.8 (93.0–98.6)	
2015	93.6 (90.5–96.8)			93.7 (90.4–97.0)		
2007–2016	94.4 (93.5–95.3)	93.0 (92.0–94.0)	89.1 (87.8–90.5)	94.6 (93.7–95.5)	93.3 (92.2–94.3)	89.4 (88.0–90.9)

*Adjusted for fixed values for age at transplant (8.9 years), male sex (61%), deceased donor (59%), glomerulonephritis (14%), CAKUT (34%), hereditary nephropathy (11%), and cystic kidney disease (8%)

Overall unadjusted patient survival after transplantation was 98.8% (95% CI: 98.4–99.3), 98.5% (95% CI: 97.9–99.0), and 97.2% (95% CI: 96.3–98.1) at 1, 2, and 5 years post-transplantation, respectively.

## Discussion

In this ESPN/ERA-EDTA Registry study, we found a stable incidence and an increasing prevalence of nearly 2% per year in European pediatric KRT over the last decade. The first mode of KRT was stable over time, with the majority of patients initiating KRT on dialysis (both HD and PD 40%), and 20% of patients received a pre-emptive kidney transplant. Patient survival was good and did not change over time, but we found a shift in causes of death. Patients who commenced KRT before 2010 died more often from infectious causes, whereas patients commencing KRT later were more likely to die from cardiovascular disease.

### Incidence

A previous ERA-EDTA Registry study reported a stable incidence for patients initiating KRT at an age below 19 years of age between 1997 and 2006 [12]. Similarly, we found a stable overall incidence of European patients commencing KRT before the age of 15 years over the last decade. Also, in Australia and New Zealand, the overall trend in the incidence of treated CKD 5 patients <18 years was stable for the past 20 years [7]. A more recent ERA-EDTA Registry study, studying the years 2001 to 2011, reported a small decrease of 2.5% per year in the incidence of European patients below 19 years [1]. Although the incidence increased prior to 2004, also in the USA, the incidence of children (0–21 years of age) requiring KRT has steadily declined between 2004 and 2016 [6]. Differences between our study and the other cohorts might in part be caused by different age limits of included patients, as well as by differences in the years studied. However, none of the recent studies reported an increase in incident pediatric patients requiring KRT for CKD 5, suggesting that pediatric nephrologists are succeeding in their efforts to prevent progression to stage 5 CKD.

In adults, lifestyle-related diseases such as hypertension, diabetes, and obesity are important risk factors for CKD [13]. Although there are some studies reporting similar associations for the pediatric population [14, 15], causes of CKD 5 in children are mostly of congenital and hereditary origin [2]. We did, however, observe changes in the causes of kidney disease over time. The number of children who needed KRT due to CAKUT decreased on average by 3.3% per year over the 10-year study period. According to the European network of congenital anomaly registries, there were more live births of children with congenital urinary anomalies in the same period [16], which might further strengthen the hypothesis of successful pre-CKD 5 treatment in this category of European children with CKD. We also found a decrease in the number of children initiating KRT due to rare diseases, such as metabolic disorders, HUS, and vasculitis, which might be the result of advances in timely and more accurate diagnosis, as well as the emergence of new therapeutics (i.e., biologics and monoclonal antibodies). At the same time, we found an increase in the number of children initiating KRT because of an unknown or missing cause of CKD 5. Although this might be due to a registration issue, it could also suggest that the number of complex cases, in which it is difficult to identify a single or clear diagnosis, accepted into KRT programs is increasing. Furthermore, it might also be the result of late referral, which might imply that fewer biopsies were performed. Indeed, in a UK study, patients with an uncertain etiology were more likely to be referred late [17].

### Prevalence

In accordance with findings in European adults [1] and in Australian children [7], this study found that the overall prevalence of European children on KRT has increased by nearly 2% per year over the 10-year study period. In contrast, the prevalence of KRT for pediatric patients from the USA and from New Zealand remained stable for the most recent decade [6, 7]. In the US, the incidence rate decreased and the survival improved resulting in a stable prevalence rate [6].

We observed an increasing trend in prevalence for all age groups and for all treatment modalities. Throughout the study period, the prevalence for both dialysis modalities was similar, whereas the number of patients living with a functioning transplant was about 4 to 5 times larger than the number of patients treated with dialysis. We did not find any further improvement in patient survival, but as long as the number of new CKD 5 cases is higher than the numbers of deaths, the prevalence rate will continue to grow.

### Mortality

Overall, 1- and 5-year patient survival on KRT were 98% and 94%, respectively, and are similar to or slightly higher than rates reported in other high-income countries [18]. We found a stable patient survival throughout the decade. Although the 1-year patient mortality has decreased by 20% over the last decade in US pediatric patients, the 5-year survival probability for those starting KRT from 2002 to 2006 was 89% [19], whereas it was 91% for those starting KRT between 2007 and 2011 [6] and therefore patient survival in the US was lower than that in Europe.

The improvement in patient survival of pediatric KRT has possibly reached its ceiling, but mortality remains at least 30 times higher than in the general pediatric population [18].

Patients initiating KRT between 2007 and 2009 died more often from infections, whereas cardiovascular disease was the leading cause of death for patients initiating KRT between 2010 and 2012. Although others also reported cardiovascular disease and infections to be the leading causes of death in pediatric KRT patients [6, 20–22], a decrease in cardiovascular mortality due to vigorous cardioprotective management has been reported. However, these findings were mainly based on data from patients treated during the last decades of the twentieth century or from adult patients who commenced KRT during childhood [20, 22–24]. Due to a high number of cases for whom the cause of death was reported as unknown or missing, as well as the relatively short period of time, we cannot draw any conclusions about trends in causes of death over time. Future studies with longer periods of follow-up are needed to determine whether a trend towards higher cardiovascular mortality exists in the European pediatric KRT population, and which specific factors are involved.

### Kidney transplantation

We did not find an overall trend in the number of kidney transplants performed. Also the graft survival remained stable over time. The prevalence of kidney transplantation increased over the decade as the incidence of kidney transplantation continues to outweigh the number of patients whose kidney graft fails. One-year graft survival remained stable over the study period ranging from 91.5 to 97.3%. Overall graft survival was 94% at 1 year and 89% at 5 years and thereby slightly higher than in registry studies from Australia and New Zealand (83%) and the USA (86% for living donor kidneys and 79% for deceased donor kidneys) [7, 25].

As with every KRT registry, a limitation of our study is that only CKD 5 patients treated with KRT are included, and the numbers presented in this study do not reflect pediatric patients with CKD 5 not (yet) on KRT. Furthermore, only countries that participated in the ESPN/ERA-EDTA Registry for the entire study period of 2007 to 2016 are included in the current study, and our study might not be fully representative for the whole of Europe. However, our study is the first one to provide data on trends in incidence, prevalence, and rates of kidney transplantation and patient survival for European pediatric KRT patients over a 10-year period.

In conclusion, we found a stable incidence and an increasing prevalence of European children on KRT over the past decade. Five-year patient survival was good and did not change over time. Also, the annual number of kidney transplantations performed did not change. These data can be used for future resource planning of pediatric KRT in Europe.

## Supplementary information

ESM 1 (DOCX 14 kb)
