# Supplementary material for: Ten-year trends in epidemiology and outcomes of pediatric kidney replacement therapy in Europe: data from the ESPN/ERA-EDTA Registry
Source: Pediatr Nephrol. 2021 Jan 22;36(8):2337–48. doi: 10.1007/s00467-021-04928-w (PMC8260419; doi:10.1007/s00467-021-04928-w)
Supplement: Supplementary file 1 — (DOCX 14 kb) [file 467_2021_4928_MOESM1_ESM.docx]

**Supplementary Material**

**Table S1** Unadjusted patient survival probabilities at one, two, and five years stratified by sex, age and treatment at KRT initiation

|  | **Unadjusted patient survival probability (95% CI)** | | |
| --- | --- | --- | --- |
|  | **One year** | **Two-years** | **Five-years** |
| **Sex** |  |  |  |
| Male | 97.8 (97.2–98.4) | 96.7 (95.9–97.4) | 94.8 (93.8–95.5) |
| Female | 97.3 (96.5–98.0) | 95.6 (95.0–96.9) | 93.9 (92.7–95.1) |
|  |  |  |  |
| **Age at KRT** |  |  |  |
| 0–4 years | 95.2 (94.1–96.3) | 92.9 (91.6–94.3) | 90.6 (89.0–92.3) |
| 5–9 years | 98.2 (97.4–99.6) | 97.4 (96.4–98.3) | 94.9 (93.3–96.4) |
| 10–14 years | 99.1 (98.7–99.6) | 98.5 (97.9–99.1) | 97.1 (96.2–98.1) |
|  |  |  |  |
| **Treatment at KRT** |  |  |  |
| HD | 97.6 (96.9–98.4) | 96.3 (95.4–97.3) | 94.2 (92.9–95.5) |
| PD | 96.9 (96.1–97.8) | 95.2 (94.1–96.2) | 92.6 (91.2–94.0) |
| Tx | 99.3 (98.8–99.9) | 99.3 (98.8–99.9) | 98.9 (98.1–99.7) |

Abbreviations: KRT, kidney replacement therapy; HD, hemodialysis; PD, peritoneal dialysis; Tx, pre-emptive kidney transplantation
